# Left-handed Cardiac Surgery: Tips from set up to closure for Trainees and their Trainers

**DOI:** 10.1186/1749-8090-10-S1-A43

**Published:** 2015-12-16

**Authors:** Clare Burdett, Joel Dunning, Andrew Goodwin, Maureen Theakston, Simon Kendall

**Affiliations:** 1 Department of Cardiothoracic Surgery, James Cook University Hospital, Middlesbrough, UK

## Background/Introduction

There are certain barriers which left-handed surgeons can face when training but these are not necessary and often perpetuated by a lack of knowledge. Most obstacles have been encountered and overcome at some point but unless recorded and disseminated they will have to be resolved repeatedly by each trainee and their trainers.

## Aims/Objectives

This review highlights the barriers faced by left-handed trainees in cardiac surgery and gives practical operative advice for both trainers and trainees to help overcome them.

## Method

We have drawn on the knowledge and experience of left-handed Consultant surgeons as well as left-handed trainees and the right-handed Consultants who train them.

## Results

We provide advice on planning and engaging the theatre team. The main components of a standard cardiac operation from opening to closure are considered as well as conduit harvest. Assisting others and emergencies are also discussed. This is followed by information on working with right and left-handed trainers.

## Discussion/Conclusion

Barriers to the progress of the left-handed trainees exist. At times they can seem insurmountable, especially when working in isolation from other left-handers. However, they can be easily removed with the co-operation of others. Both right and left-handers can make good trainers. Providing information on proven left-handed training practices will accelerate the process and enhance training.

